# Reactive Oxygen Species and Pulmonary Vasculature During Hypobaric Hypoxia

**DOI:** 10.3389/fphys.2018.00865

**Published:** 2018-07-09

**Authors:** Patricia Siques, Julio Brito, Eduardo Pena

**Affiliations:** Institute of Health Studies, Arturo Prat University, Iquique, Chile

**Keywords:** reactive oxygen species, pulmonary hypertension, hypobaric hypoxia, NADPH oxidase, pulmonary vasculature

## Abstract

An increasing number of people are living or working at high altitudes (hypobaric hypoxia) and therefore suffering several physiological, biochemical, and molecular changes. Pulmonary vasculature is one of the main and first responses to hypoxia. These responses imply hypoxic pulmonary vasoconstriction (HPV), remodeling, and eventually pulmonary hypertension (PH). These events occur according to the type and extension of the exposure. There is also increasing evidence that these changes in the pulmonary vascular bed could be mainly attributed to a homeostatic imbalance as a result of increased levels of reactive oxygen species (ROS). The increase in ROS production during hypobaric hypoxia has been attributed to an enhanced activity and expression of nicotinamide adenine dinucleotide phosphate oxidase (NADPH oxidase), though there is some dispute about which subunit is involved. This enzymatic complex may be directly induced by hypoxia-inducible factor-1α (HIF-1α). ROS has been found to be related to several pathways, cells, enzymes, and molecules in hypoxic pulmonary vasculature responses, from HPV to inflammation, and structural changes, such as remodeling and, ultimately, PH. Therefore, we performed a comprehensive review of the current evidence on the role of ROS in the development of pulmonary vasculature changes under hypoxic conditions, with a focus on hypobaric hypoxia. This review provides information supporting the role of oxidative stress (mainly ROS) in the pulmonary vasculature’s responses under hypobaric hypoxia and depicting possible future therapeutics or research targets. NADPH oxidase-produced oxidative stress is highlighted as a major source of ROS. Moreover, new molecules, such as asymmetric dimethylarginine, and critical inflammatory cells as fibroblasts, could be also involved. Several controversies remain regarding the role of ROS and the mechanisms involved in hypoxic responses that need to be elucidated.

## Introduction

There are two main sources or conditions of hypoxia to which humans are exposed: normobaric hypoxia (at sea level) and hypobaric hypoxia (at high altitudes). Exposure to hypobaric hypoxia can be classified as acute, chronic hypoxia (CH) or chronic intermittent hypoxia (CIH) exposure (Richalet et al., 2002). High-altitude or hypobaric hypoxia exposure leads to a reduction in arterial oxygen saturation due to a drop in the partial pressure of oxygen (PaO_2_), triggering several physiological and/or pathological effects, with pulmonary vascular system changes being among the most important effects (Jensen et al., 1992; Moudgil et al., 2005; Brito et al., 2007). These changes are dependent on exposure time and altitude (Scherrer et al., 2013).

In the clinic, the most well-known CIH is obstructive sleep apnea (OSA), with a prevalence of approximately 14% of the general population. In this condition, the hypoxic state is intermittently maintained for brief periods (Dumitrascu et al., 2013). Although less common, a large body of literature is available on hypobaric hypoxia, which results from living at or ascending to a high altitude. Acute exposure typically applies to tourists and recreational climbers, whereas CH exposure applies to people permanently living at high altitude. Both conditions and their related diseases are rather well characterized (León-Velarde et al., 2005). Over 100 million people are estimated to live at high altitude (Niermeyer et al., 1995; Moore, 2001).

A new model of CIH has been described after the development of mine settlements at high altitudes (over 3000 masl), although this model of hypobaric hypoxia is completely different from other types of intermittent hypoxia such as OSA (Richalet et al., 2002). This type of exposure affects workers commuting to work at high altitude for several days and then resting at sea level for the same period over several years (Richalet et al., 2002). This condition is rather new, and few research studies on the subject are available. Despite some similarities, the changes and mechanisms involved may not be applicable to all types of intermittent hypoxia.

The first vasculature pulmonary phenomenon is hypoxic pulmonary vasoconstriction (HPV) in response to alveolar oxygen pressure. This intrinsic mechanism in the lungs optimizes systemic oxygen delivery by matching perfusion to ventilation (Von Euler and Liljestrand, 1946; Desireddi et al., 2010; Dunham-Snary et al., 2017). This vasoconstrictor effect is modulated by vasoactive substances present in the blood or released from the endothelium and lung parenchyma and can vary with age and species (Leblanc et al., 2013). In contrast, in the systemic vasculature, hypoxia causes a vasodilator effect through the ATP-dependent potassium channel, leading to the relaxation of smooth muscle cells (SMCs) (Weir and Archer, 1995). When alveolar hypoxia is sustained over time, as in CH or in patients with chronic lung disease, HPV can contribute to initiating vascular remodeling and the subsequent development of pulmonary hypertension (PH) and, ultimately, heart failure (Peñaloza et al., 1971; Xu and Jing, 2009; León-Velarde et al., 2010; Rimoldi et al., 2012).

The pathological mechanism of HPV-induced PH involves a wide array of mechanisms and pathways. There is growing evidence that reactive oxygen species (ROS) increase as a result of hypoxia exposure and could play a key role in the pulmonary vasculature responses to hypoxia (Guzy and Schumacker, 2006). ROS can impair the activity of vasodilators or their production and can also elicit inflammatory processes leading to a local inflammatory environment in the pulmonary vasculature (Aggarwal et al., 2013; Wong et al., 2013). Moreover, the molecular mechanism in the pulmonary vasculature that is involved in the response to hypoxia depends on the time and type of exposure and on genetic and individual variabilities (Beall, 2006; Simonson et al., 2010; Scherrer et al., 2013).

For these reasons, this review attempts to provide comprehensive insight into the main factors, sources and mechanisms in the interactions between ROS and other cellular components in the development of vascular responses or diseases due to hypoxia and/or other similar stressors, with an emphasis on pulmonary vasculature under hypobaric hypoxic conditions.

## Hypoxic Pulmonary Vasoconstriction, Remodeling, and ROS

As mentioned above, HPV is the main and unique response to hypoxia exposure, whose core mechanism resides in calcium flux. Interestingly, in turn, HPV is closely intertwined with ROS activity to elicit this critical response. Therefore, Ca^2+^ plays a fundamental role in PA vasoconstriction (Dunham-Snary et al., 2017). However, whether the internal release of calcium (Gelband and Gelband, 1997) or the influx of extracellular calcium (Weissmann et al., 2006) triggers SMCs contraction is currently debated in the literature.

Thus, it is important to elucidate the role of ROS in the molecular mechanism underlying the increase in the intracellular calcium concentration ([Ca^2+^]_i_). Studies have shown that hypoxia-induced increases in [Ca^2+^]_i_ in pulmonary artery SMCs (PASMCs) require an increase in ROS signaling, specifically those involving super oxide anion (O_2_^∙-^) and hydrogen peroxide (H_2_O_2_) (Jefferson et al., 2004). Thus, oxidative signaling via ROS potentially triggers a Ca^2+^ release from the sarcoplasmic reticulum (SR), allowing extracellular calcium to enter through voltage-dependent calcium channels (VDCCs) and allowing capacitive calcium entry through store-operated calcium channels, thereby resulting in contraction (Wang et al., 2004). Notwithstanding, the main source of calcium in this process remains unclear (Staiculescu et al., 2014). However, studies have shown that extracellular calcium influx caused possibly by hypoxia-induced ROS signaling is necessary to elicit the HPV response since removal of extracellular calcium ions abolishes the hypoxia-induced increase in [Ca^2+^]_i_ in SMCs (Weir and Archer, 1995; Desireddi et al., 2010).

A sustained HPV could contribute to starting a vascular remodeling and the subsequent development of PH (Peñaloza et al., 1971; León-Velarde et al., 2010; Rimoldi et al., 2012). Under physiological conditions, the thickness of the vascular wall is maintained by a fine balance between cellular proliferation and apoptosis (Pak et al., 2007; Welsh and Peacock, 2013). If this balance is disrupted in favor of decreased apoptosis and increased proliferation, the vascular wall thickens and can eventually obstruct the vessel lumen. This structural process, which leads to augmented resistance, is known as vascular remodeling (Kato and Staub, 1966). This remodeling effect can be rapidly initiated (Jensen et al., 1992; Moudgil et al., 2005; Engelhardt et al., 2014). In addition, HPV has been documented in several animal models, including fetal lambs, newborn piglets, rats, and mice (Wong et al., 2013).

This imbalance, which might be triggered by oxidative stress in favor of proliferation, occurs in PAs via different processes: an inhibition of antimitogenic factors (e.g., NO and prostacyclin) and increased synthesis and release of different mitogenic stimuli [endothelin-1 (ET-1), 5-hydroxytryptamine platelet-derived growth factor (PDGF) and vascular endothelial growth factor (VEGF)]. There is also a third relevant mechanism: an increased production of extracellular matrix components (Stenmark and Mecham, 1997) through the release of inflammatory mediators [interleukin (IL)-6, IL-8, IL-22, signal transducer and activator of transcription 3 (STAT3), nuclear factor of activated T-cells, stromal cell-derived factor-1, monocyte chemoattractant factor-1 (Kishimoto et al., 2015) and inducible-nitric oxide synthase (iNOS) (Visser et al., 2010; Bansal et al., 2013), among others]. It is noteworthy that not all inflammatory mediators participate in remodeling or PH such as IL-4/IL-13, STAT6, and TLR-MyD88 (El Kasmi et al., 2014).

### Controversial Effects of HPV and ROS on Potassium Channels (K^+^ Channels)

Although redox signaling has been suggested to be a crucial event in HPV and remodeling, whether an increase or decrease in ROS triggers these processes has been debated (Waypa et al., 2002). First, studies have noted that hypoxia decreases the generation of ROS in PASMCs and shifts the cytosol into a more reduced state, which alters the redox-sensitive thiol groups in the regulatory subunits of Kv channels (Kv2.1), causing the channels to close and altering the resting membrane potential (E_m_) in PASMCs. Membrane depolarization then leads to voltage-gated (L-type) calcium channel opening followed by an influx of extracellular calcium into the cytoplasm, resulting in vasoconstriction (Weir and Archer, 1995; Archer et al., 1998). These results are derived from studies on isolated PASMCs, thus simulating a hypoxic environment with powerful reducing agents, such as sodium dithionite, and the subsequent response in potassium currents (hypoxic inhibition of potassium current) may be specific for PASMCs rather than systemic artery SMCs. However, the same studies have shown that the use of sodium dithionite (an oxygen tension-reducing agent) increases the production of superoxide anions and hydrogen peroxide as much as it reduces oxygen tension (Dunham-Snary et al., 2017). Although HPV is an intrinsic response of pulmonary vasculature when there is a K^+^ channel blockage, both vasculatures respond with a vasoconstriction (Post et al., 1992). Thus, the most likely explanation of the heterogenic response in systemic and pulmonary vasculature, under hypoxia, would lie in the different cellular mechanisms induced by hypoxia itself (Dunham-Snary et al., 2017). The mentioned discrepancies regarding whether hypoxia leads to an oxidized state with high ROS levels or a reduced state with low ROS levels may be explained by the diversity of methodology or models, including type of artery, severity and type of hypoxia, time of exposure, pH and PCO_2_ disturbances, and type of cellular layer.

## Oxidative Stress, ROS, and Hypoxia

Oxidative stress is a condition that occurs when there is an increase in ROS at the cellular level. ROS represent a set of reactive species that are derived from oxygen that have one or more unpaired electrons in their outer orbital layer, which make them very unstable and highly reactive. For these reasons, ROS can bind to and oxidize different cellular compounds (lipid, DNA, protein, and cellular membrane, among others), thereby modifying their structure and function and activating cellular signaling (Jefferson et al., 2004; Montezano and Touyz, 2012). Known free radicals include superoxide anion (O_2_^∙-^), hydroxyl ion (OH), peroxyl (RO_2_), and alkoxy agents (RO^.^). Certain non-radicals that are either oxidizing or easily converted into oxidative elements include hypochlorous acid (HOCl), ozone (O_3_), singlet oxygen (^1^O_2_), and hydrogen peroxide (H_2_O_2_). Notably, there are other oxidative ions that contain nitrogen and can contribute with a nitrosative stress under the name of reactive nitrogen species. The most representative species is peroxynitrite ion (ONOO^-^), which is derived from NO and O_2_^∙-^ binding (Radi et al., 1991; Beckman and Koppenol, 1996).

Despite the apparent paradox whereby, in a hypoxic environment, there is more oxidative stress, which has generated a long debate, it is currently agreed that hypoxia and consequently PH are associated with a high level of oxidative stress (Bowers et al., 2004; Guzy and Schumacker, 2006). Several studies have provided evidence of this phenomenon. Using F_2_-isoprostane levels in urine as a sensitive biomarker of oxidative stress (Dorjgochoo et al., 2012), ROS levels were observed to be two- to threefold higher in PH patients than in control subjects (Wong et al., 2013). Likewise, higher plasma concentrations of malondialdehyde (MDA), a compound derived from oxidative stress-induced lipid oxidation, have been reported in patients with idiopathic pulmonary arterial hypertension (Irodova et al., 2002) and in rats under hypobaric hypoxia (Lüneburg et al., 2016). Similarly, in endothelial cells, increased levels of 8-hydroxy-deoxyguanosine (8-OHdG), a marker of deoxyribonucleic acid oxidation, have also been found (Bowers et al., 2004; Kishimoto et al., 2015). Thus, an increase in ROS might lead to inflammation, hypertrophy, cellular proliferation, apoptosis, migration, and fibrosis (Wong et al., 2017) while also generating an endothelial dysfunction (Montezano and Touyz, 2012), all of which are components of the remodeling process, which will ultimately manifest in a PH. Therefore, ROS imbalance as a result of hypoxia may be strongly involved in the hypoxic response of pulmonary vasculature at any hypoxic condition, and restoring this balance has prompted several studies.

### Main Sources of ROS in the Vascular System Under Hypoxic Conditions

Reactive oxygen species can be produced as a sub-product of the electron transporter chain (ETC) in the mitochondria. The ETC is composed of four mega-complexes that mediate the transfer of electrons down a redox potential gradient through a series of carriers, resulting in the acceptance of an electron by O_2_ and producing ATP and water (Moudgil et al., 2005). A small percentage of total electron flux involves unpaired electrons, resulting in the generation of ROS, notably superoxide radicals, within the mitochondrion. To remove this latter ROS, mitochondria express a unique, inducible isoform of superoxide dismutase (SOD2, manganese SOD or MnSOD). MnSOD transforms toxic superoxide radicals into H_2_O_2_, which can in turn serve as a diffusible redox mediator in HPV (Archer et al., 1993). Interestingly, the release of superoxides through enzymes and anion channels within the mitochondria of PASMCs can be modulated by diffusible H_2_O_2_ (Moudgil et al., 2005). Additionally, the relationship between mitochondrial ROS and PA remodeling in response to CH is supported by the observation that depletion of Rieske iron-sulfur protein (RISP), a mitochondrial complex III protein that is required for ROS generation, in mouse ECs and SMCs prevents CH-induced PH (Guzy and Schumacker, 2006; Waypa et al., 2016).

Increasing evidence suggests that the principal source of ROS in the cardiovascular system is the enzymatic complex nicotinamide adenine dinucleotide phosphate oxidase (NADPH oxidase) (Moudgil et al., 2005). The NADPH oxidase complex is structurally composed of transmembrane and cytosolic proteins that use NADPH as the electron donor to reduce molecular oxygen to O_2_^∙-^ and H_2_O_2_. Studies have characterized seven members of the NOX family of NADPH oxidases (NOX1–5 and dual oxidase 1 and 2), which are expressed within specific tissues and organs (Bache and Chen, 2014), including neurons, skeletal muscle, myocytes, hepatocytes, ECs, hematopoietic cells, stem cells, and cardiomyocytes (Yu et al., 2016). However, NOX4 has been reported as the most prevalent subunit in the cardiovascular system (Chen et al., 2012).

The oxidase role of NADPH has been further supported using animal models with antioxidant overexpression such as catalase or glutathione peroxidase-1 (GP_x1_). Further, experimental pharmacologic intervention with antioxidants and anti-inflammatory products (probucol, *N*-acetylcysteine, tempol, erdosteine, allicin, pyrrolidine dithiocarbamate, superoxide dismutase, allopurinol, sulfur dioxide, resveratrol, hydrogen water, and EUK-134) have been demonstrated to ameliorate or inhibit PH and right ventricular dysfunction (Waypa et al., 2002; Wong et al., 2013; Kishimoto et al., 2015). However, it is important to highlight that clinical studies have shown inconclusive results using pharmacological or dietary antioxidant treatments, despite the ample evidence on the importance of ROS in experimental models. It is likely that the precise mechanism of ROS and the pharmacokinetics of antioxidants in humans are still not well understood in hypoxic diseases (Wong et al., 2013).

### The Oxidase Role of NADPH

There is evidence of increased mRNA and protein expression of a specific NADPH isoform, NOX4, in the lungs of rats exposed to chronic hypobaric hypoxia, presenting PH, in which the concentration of MDA is increased by up to twofold after CIH or CH exposure (Lüneburg et al., 2016). In agreement with this evidence, silencing of NOX4 and p47phox (another subunit of NADPH oxidase) has been shown to attenuate oxidative stress and human and rat PASMC proliferation (Mittal et al., 2012). In addition, both chronic and intermittent hypoxia leads to a notable increase in superoxide radicals, which can impair and decrease NO bioavailability (Barman et al., 2014; Siques et al., 2014).

In contrast, recent studies have shown that NOX4 does not influence the development of hypoxia-induced pathologies in the lung such as HPV or PH (Veith et al., 2016). Supporting the above findings, augmented superoxide anion (O_2_^∙-^) production has been detected in mice with NOX2- and NOX1-overexpressing SMCs. NOX1- and NOX2-derived O_2_^∙-^ may contribute to the formation of ONOO^-^ and subsequently to vascular dysfunction, while NOX4 overexpression increases H_2_O_2_ formation (Martyn et al., 2006; Serrander et al., 2007; Veith et al., 2016). The reason for the latter resides in the fact that NOX4 may be incapable of scavenging NO in the PA due to a rapid dismutation of O_2_^∙-^ to H_2_O_2_ before its release from the enzyme (Takac et al., 2011). Nevertheless, NOX4-produced H_2_O_2_ can also elicit different effects depending on its concentration, ranging from a beneficial effect on endothelial cell activity in PAs at low H_2_O_2_ concentrations to the development of SMC hypertrophy at high H_2_O_2_ concentrations (Schröder et al., 2012; Veith et al., 2016). Therefore, the precise role of each of the NADPH subunits in hypoxia appears to be controversial. However, until recently, NOX4 was considered to be the most relevant according to several authors (Sturrock et al., 2006; Mittal et al., 2007; Nisbet et al., 2009). Drawing a definite conclusion will require further research.

## Pulmonary Arterial Layers, Fibroblasts, and ROS in Hypoxia

### Adventitial Cells

Hypoxia induces a more than twofold increase in adventitial thickness in PAs via both fibroblast hypertrophy and hyperplasia and an increase in the surrounding collagen matrix (Meyrick and Reid, 1979). A recent study in rats under CIH showed increased the cellularity of inflammatory cells without changes in lumen size as distinctive features of this condition (Brito et al., 2015). The main trigger or regulator of these processes would be ROS (Welsh et al., 2001). Under hypoxic conditions, fibroblasts appear as key cells as a result of their capacity to migrate into the medial layer and transform into SMCs (Stenmark et al., 2002), thus contributing to vascular remodeling. Moreover, resident adventitial cells could also be activated and reprogrammed for several different behaviors, participating in remodeling and SMC tone (Welsh and Peacock, 2013).

Thus, under hypoxic conditions, adventitial fibroblasts would also act via the generation of matrix proteins (Sartore et al., 2001), which contribute to the narrowing of the vascular lumen, via cytokines, growth factors, and inflammatory mediators [IL-6, STAT3, hypoxia-inducible factor-1 (HIF-1), and C/EBPβ]. The last one has been shown to be paracrine regulators of vascular macrophages for chronic inflammation, remodeling, and PH (El Kasmi et al., 2014).

The molecular mechanism by which hypoxia stimulates proliferation in PA fibroblasts is under study. Nevertheless, there is evidence that mitogen-activated protein (MAP) kinase, Erk1/2 and related stress-activated kinases, such as Jnk and p38 MAP kinase, are key regulators of cell proliferation and can be activated in response to hypoxic stress (Seko et al., 1996; Robinson and Cobb, 1997; Das et al., 2001). Further support for the role of hypoxia-induced oxidative stress in the activation of these protein kinases has been described (Dworakowski et al., 2006; Schröder et al., 2009; Montezano and Touyz, 2012; Yamada et al., 2012). In addition, recent studies show that HIF-1α stabilization in all PA cells elicits the production of ROS via p22phox-containing NADPH oxidase. Therefore, HIF-1α-induced ROS production might contribute to the activation of PA fibroblasts and could be another mechanism by which hypoxia induces PA fibroblast proliferation (Welsh et al., 2001). Controversially, a recent cell culture study reported that ROS levels in human fibroblasts decrease under hypoxic conditions (Sgarbi et al., 2017); however, this finding requires further verification.

Another mechanism for fibroblast proliferation under hypoxia would involve their capacity to transdifferentiate into other cell types such as myofibroblasts through a complex network of microenvironmental factors and extracellular matrix components (Stenmark et al., 2002).

### Smooth Muscle Cells

It is well known that medial thickness greatly determines pulmonary vascular resistance. The pre-capillary segment of the pulmonary vascular bed contributes to pulmonary vascular resistance and determines PA pressure. Under hypoxic conditions, these vessels enhance their muscularization (Meyrick and Reid, 1979), representing a key feature of hypoxic pulmonary vascular remodeling (Pak et al., 2007). This effect has been observed in CH (Penaloza and Arias-Stella, 2007) and CIH, but to a lesser extent (Brito et al., 2015). Whether acute hypoxia has proliferative effects on PASMCs is controversial, though several studies have demonstrated either increased ROS levels or cellular proliferation in acute hypoxia (Wedgwood et al., 2001).

Vascular SMCs play important roles in the physiological regulation of vascular tone and vascular remodeling. The mechanisms by which hypoxia lead pulmonary vessels to constrict and coronary vessels to relax are subject to ongoing research. Hypoxia-induced closure of Kv channels could be the result of a change in cytoplasmic ROS or redox status in PASMCs. Moreover, NADPH oxidase has been shown to bind the β-subunit of Kv channels, thus supporting a link between ROS and K^+^ channels and cellular excitability (Wu et al., 2007).

Notably, ET-1 is upregulated during hypoxia and has been demonstrated to influence SMC proliferation via NADPH oxidase-derived superoxides and to contribute to the pathogenesis of hypoxic-induced PH (Madden et al., 1992; Li et al., 1994; Liu et al., 2007). Moreover, administration of ET-1 significantly increases ROS production in both PASMCs and coronary artery SMCs (Wu et al., 2007), whereas inhibition of NADPH oxidase in bovine fetal PASMCs activates the apoptosis process and avoids ET-1-mediated SMC proliferation (Wu et al., 2007).

The role of ROS SMC proliferation is also a controversial issue. While one study has shown that acute hypoxia significantly reduces superoxide production in both human PASMCs and coronary arterial SMCs (Gupte and Wolin, 2006), others have demonstrated increased ROS production in pulmonary arteries due to acute hypoxia and CIH (Wedgwood et al., 2001; Schumacker, 2011; Siques et al., 2014). There are several explanations for the lack of superoxide: a differential regulation of NADPH oxidases due to higher basal cytosolic NADPH oxidase levels in PAs (Gupte and Wolin, 2006), a subcellular localization of NOX, which may be critical for the activation of downstream signaling, and differences in SOD activity between PASMC and coronary artery SMCs. Regrettably, the available literature does not allow a definitive explanation.

Another possible pathway involved in SMC proliferation could be explained by ROS-activated p38MAPK, as demonstrated in hypoxia-induced proliferation and contraction in rat PAs (Karamsetty et al., 2002). Remarkably, this latter effect has not been observed in systemic vasculature (Sigaud et al., 2005). In addition, carbonylation of annexin A1 may contribute to the development of vascular remodeling and PH since ET-1 and serotonin (5-HT) in PASMCs could promote the carbonylation of annexin A1 protein (Wong et al., 2008). Annexin A1 exerts anti-inflammatory, antiproliferative, and proapoptotic effects. Consequently, inhibition of protein carbonylation may be a novel therapeutic target for hypobaric hypoxia PH (Wong et al., 2013).

### Endothelial Cells

The EC layer is in a unique position that allows it to respond to circulatory and blood factors, acting as a vasculature integrator, modulator, and signal transducer via paracrine signaling (Furchgott and Zawadzki, 1980).

Under hypoxic conditions, an endothelial dysfunction occurs that not only alters the vascular tone but also contributes with inflammatory and proliferative mediators, ultimately leading to vascular remodeling (Griendling and Ushio-Fukai, 2000). Thus, ECs regulate the vascular tone and pulmonary remodeling by producing vasodilator and antiproliferative NO or vasoconstrictive pro-proliferative factors (ET-1, angiotensin II, thromboxane A_2_ and PDGF-B; Pak et al., 2007) that are exclusive to ECs but not to SMCs (Ambalavanan et al., 1999).

A main endogenous vasodilator and antiproliferative is NO, which is synthetized in ECs by endothelial nitric oxide synthase (eNOS), through stimulation of soluble guanylyl cyclase (sGC) to increase the level of cyclic GMP in SMCs (Förstermann et al., 1986). Therefore, lesser NO availability would lead to endothelial dysfunction (Cai and Harrison, 2000; Wolin et al., 2010; Frazziano et al., 2012). Increased ROS levels under hypoxia have been widely demonstrated during this review and would play a key role in impairing NO production and bioavailability (Cai and Harrison, 2000; Siques et al., 2014). The described mechanisms through which ROS would alter NO levels in ECs include NO degradation, ONOO^-^ production, and increasing oxidative stress (Radi et al., 1991; Millatt et al., 2003; Sun et al., 2010; Dumitrascu et al., 2013), stimulation of uncoupled eNOS formation (BH4 decreased) (Sun et al., 2010), stimulation of iNOS activity and enhancement of O_2_-production (Loscalzo, 2001).

Interestingly, enhanced associations between iNOS and asymmetric dimethylarginine (ADMA) level leading to airway inflammation and also in the vasculature have been described (Wells and Holian, 2007; Visser et al., 2010). ADMA induces a decrease in endothelium-dependent vasodilation through the inhibition of eNOS (Böger, 2006; Wilcken et al., 2007). ADMA also interacts with ROS in the vasculature by disturbing the usual flow of electrons between the two domains of eNOS, becoming a generator of superoxide radicals instead of NO (Sydow and Münzel, 2003). Recently, it has been demonstrated that exposure to CIH and CH induces a steady increase of ADMA in human (Lüneburg et al., 2017) and rat models (Lüneburg et al., 2016). Therefore, this mechanism could also contribute to the hypoxic inflammatory status, hypoxic endothelial dysfunction, and subsequent vascular remodeling.

A limitation of this review is that it focuses mainly on hypobaric hypoxia, which is the hypoxia experienced at high altitude, but it was also necessary to include studies conducted in either simulated hypobaric or normobaric hypoxia. This could introduce some bias because it has been well determined that different responses can occur according to the source of the hypoxia (Savourey et al., 2003; Bonetti et al., 2009; Debevec and Millet, 2014).

## Conclusion

This review provides information supporting the role of oxidative stress (mainly ROS) in the pulmonary vasculature’s responses under hypobaric hypoxia and depicting possible future therapeutic and research targets. NADPH oxidase-produced oxidative stress is highlighted as a major source of ROS. Moreover, new molecules, such as ADMA, and critical inflammatory cells as fibroblasts, could be also involved. Several controversies still remain regarding the roles of ROS and the mechanisms involved in hypoxic responses. Despite the latter, these mechanisms, independently or cooperatively, could contribute to the responses of the pulmonary vasculature to hypoxia, ultimately resulting in PH.

**FIGURE 1:**
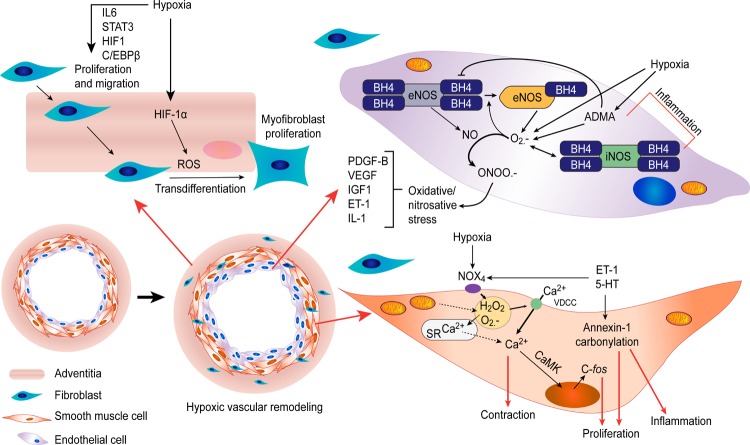
Proposed scheme: the most feasible and hypothetic mechanisms involved in the relationship between ROS and pulmonary vascular cells under hypobaric hypoxic conditions are summarized. Ca^2+^, calcium; ROS, reactive oxygen species; SR, sarcoplasmic reticulum; 5-HT, serotonin; ET-1, endothelin-1; IL-6, interleukin 6; IL-1, interleukin 1; STAT3, signal transducer and activator of transcription 3; HIF-1, hypoxia-inducible factor 1; C/EBPβ, CCAAT/enhancer-binding protein beta; PDGF-B, platelet-derived growth factor subunit B; VEGF, vascular endothelial growth factor; IGF-1, insulin-like growth factor; ONOO^-^, peroxynitrite; ADMA, asymmetric dimethylarginine; eNOS, endothelial nitric oxide synthase; iNOS, inducible nitric oxide synthase; BH4, tetrahydrobiopterin; VDCCs, voltage-dependent calcium channels; O_2_^∙-^, superoxide anion; H_2_O_2_, hydrogen peroxide; CaMK, Ca^2+^/calmodulin-dependent protein kinase.

Finally, a schematic summarizing the most feasible and hypothetic mechanisms involved in the relationship between ROS and pulmonary vascular cells under hypobaric hypoxic conditions, as a result of this review, is provided in **Figure 1**.

## Author Contributions

PS, JB, and EP equally contributed to the design of the review, gathering of information, analysis of literature, and drafting of the manuscript. All of the authors approved the final manuscript and agreed to be accountable for all aspects of the work.

## Conflict of Interest Statement

The authors declare that the research was conducted in the absence of any commercial or financial relationships that could be construed as a potential conflict of interest.
